# Improving transitions of care for complex pediatric trauma patients from inpatient rehabilitation to home: an observational pilot study

**DOI:** 10.1186/s13037-015-0078-1

**Published:** 2015-10-15

**Authors:** Susan E. Biffl, Walter L. Biffl

**Affiliations:** Children’s Hospital Colorado, 13123 East 16th Avenue B285, Aurora, CO 80045 USA; Denver Health and Hospital, Denver, CO USA; University of Colorado School of Medicine, Aurora, CO USA

**Keywords:** Post-discharge phone call, Transitions of care, Pediatric rehabilitation

## Abstract

**Background:**

Patients requiring inpatient pediatric rehabilitation following trauma or disabling illness often require complex care after hospital discharge. The patients and their families are at risk for loss of continuity of care and increased stress which can adversely affect functional and medical outcomes. This pilot study assesses the complexity of need and difficulty with obtaining services at the time of transition from inpatient to outpatient care for pediatric rehabilitation. Additionally we explored the intervention of a post discharge phone call from an experienced rehabilitation nurse to address any issues identified in this period.

**Methods:**

A rehabilitation nurse made scripted post discharge phone calls to patients and families 1–2 weeks after discharge from inpatient pediatric rehabilitation inquiring about medical appointments, medications, therapies, adaptive equipment and transition back to school. Results were recorded by the nurse then analyzed and tabulated by a rehabilitation physician.

**Results:**

Eighty two percent of patients had needs in 4–5 of the areas assessed as part of their discharge recommendations. Eighty four percent of those families contacted had difficulty with at least one area at discharge. In all cases of confusion or difficulty with the recommendations, the nurse was able to provide needed guidance to ameliorate the situation.

**Conclusions:**

This pilot study indicates that pediatric rehabilitation patient require complex care as they transition to an outpatient setting. There is significant confusion and families often have difficulty obtaining necessary care in an efficient and effective way during this transition. A post discharge phone call from an experienced rehabilitation nurse could address most of the issues that arise during the transition. This pilot study indicates a need for more investigation into interventions to improve the transition process for pediatric rehabilitation patients and suggests a post discharge phone call program could be useful intervention for pediatric rehabilitation patients and other patient populations requiring complex care such as polytrauma patients.

## Background

Pediatric patients who suffer severe trauma or disabling illness will often benefit from intensive inpatient pediatric rehabilitative care to optimize function. In addition to therapies and rehabilitative care, many of these patients have required complex care from multiple services during the course of their inpatient care, including providers from trauma, orthopedic and neurosurgery, infectious disease, neurology, ophthalmology and other subspecialties. At the time of discharge, many have multiple issues that require ongoing care and follow up. Frequently these children were healthy and only required routine well-child care prior to their injury or illness. Often their families are overwhelmed by coordinating their new complex care. At the same time the families are also coping with a newly disabled child and, especially in the setting of trauma, may be dealing with injuries and health care needs of their own. Thus the transfer from inpatient rehabilitation to home is often overwhelming and families can feel abandoned by the health care system. Necessary treatment, support and equipment can be missed or delayed. The literature supports significant challenges to pediatric rehabilitation patients and their families during this transition especially among children who have suffered brain injury [1–4].

Post discharge follow up phone calls have been utilized to improve communication with patients and families around the time of discharge from inpatient care in a variety of patient populations [5–11]. Positive outcomes of these programs that have been reported in the literature have included decreases in medication errors and emergency room visits, improved compliance with treatment plans and higher patient satisfaction. In some cases they have also decreased the need for post-discharge follow-up visits and have identified issues that required prompt care; moreover, in some cases they could ameliorate the situation during the phone call [5–11]. These types of interventions have proven to be cost effective [12]. Phone call follow-ups have not been reported for the pediatric rehabilitation population to our knowledge and have not been widely implemented.

Our multidisciplinary team sought to improve our patients’ and families’ transitions of care. The purpose of this pilot project was to document the needs of our patients and families in key areas during the discharge process, and to determine whether a post-discharge phone call would be able to meet some of those needs.

## Methods

Our pediatric rehabilitation unit is located in a metropolitan tertiary care children’s hospital that is a regional Level 1 pediatric trauma center. Each year our pediatric inpatient rehabilitation team provides care for approximately 120 pediatric rehabilitation patients and their families. We noted that when we saw patients at their 1 month follow up rehabilitation medicine visit there was often confusion and lack of follow up on recommended care. Based on this experience we hypothesized that the care our patients required upon discharge was quite complex and that many of the families had difficulties obtaining the care they required during that transition. To further investigate this issue we designed a prospective population-based quality improvement pilot project.

We designed a script for a follow up phone call to be made by an experienced rehabilitation nurse within 1–2 weeks of the patient’s discharge from the rehabilitation unit. Over the 12 month period of the 2012 calendar year, patients discharged from the inpatient pediatric rehabilitation unit were called and the responses to the scripted questions were recorded in their medical record. Diagnoses were noted. Patients were not stratified by age, race or ethnicity.

During the scripted phone call from the nurse, families were asked about 5 areas of need. These areas included: medical appointments, medications, therapies, adaptive equipment including orthotics and education including preschool and daycare settings as well as school. Our team had identified these key areas as the most common areas of post discharge care recommendations for pediatric rehabilitation patients. The inpatient rehabilitation discharge summaries were reviewed by the nurse for the recommendations in each of these areas prior to the phone call. Outcome measures collected included needs in each of these area and difficulties obtaining needed care and equipment encountered as expressed by the family. A need in each area could be identified by the treatment team in the discharge summary and orders or identified by the family in the follow up phone call. Difficulties were recorded based upon family response. If there were difficulties related to obtaining necessary care or equipment, the nurse would address the issue and document the intervention. A rehabilitation physician reviewed the documentation of the phone calls and tabulated according to whether a need was present and whether it was met and if there was an intervention by the nurse and if it was successful. Descriptive statistics were utilized to analyze the data using Microsoft Excel. This project was approved by the hospital Quality Improvement oversight board.

## Results

Responses were obtained from 65 patient families representing 56 % of those hospitalized during the study period.

**Fig. 1:**
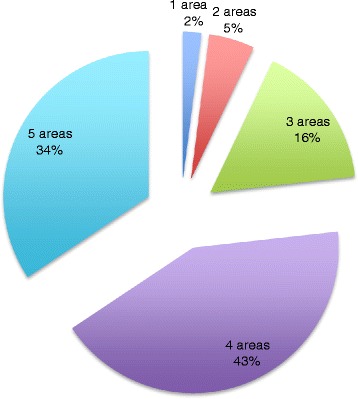
Complexity of care: Breakdown of number of areas of need per patient post discharge from inpatient rehabilitation

**Fig. 2:**
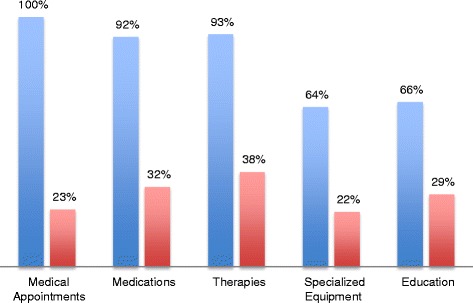
Areas of difficulty: Blue columns represent percentage of patients who require a given service and red columns represent percentage of patients who require the service who encountered difficulty obtaining needed care in that area post discharge

In 76 % of these patients, needs were identified in 4–5 of the areas studied according to the treatment plan and family report (Fig. 1). In examination of each area individually, 100 % of patients required medical appointments, 92 % of patients required medications, 93 % of patients required therapies, 64 % of patients required specialized equipment, and 66 % of patients were returning to a school or daycare setting with significantly altered functional abilities (Fig. 2).

In 84 % of families contacted, difficulties were identified in obtaining needed treatment or resources or confusion was noted regarding recommendations in these areas of their outpatient treatment plan. In those requiring medical appointments, 23 % of patients had difficulty making appointment or were unsure of which specialists they needed to see. In those requiring medications, 32 % had difficulty obtaining medications, issues with side effects or were unclear on dosing or timing of medications. In those requiring therapies, 38 % had difficulty making appointments or finding providers who were able to meet their needs. In those requiring specialized equipment, 22 % had difficulty obtaining or using the equipment. In those returning to school or daycare, 29 % had difficulty with the transition, usually related to school or daycare staff being unaware of new needs (Fig. 2).

**Fig. 3:**
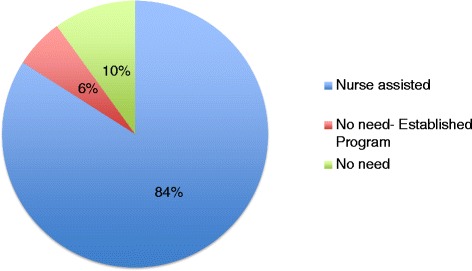
Assistance during phone call: Blue represents patients who required and received assistance during their call, green represents routine rehabilitation patients who did not require assistance during the call, red represents rehabilitation patients who were involved in established programs who did not require assistance during the call

Of note, among those patients who did not report difficulty or confusion, 40 % were involved in a patient population specific system of care such as the Neuro-Oncology, Hemispherectomy Surgery or Dorsal Rhizotomy Programs with established structured follow up support or had a planned readmission for a rehabilitation “tune-up” (Fig. 3).

In all instances of confusion or difficulty identified, the nurse was able to provide information to the family that enabled them to address the situation. There were no complaints or negative comments from families regarding the post-discharge phone call.

## Discussion

Transitions of care are times when there is increased risk of confusion and mis-communication which can result in lack of necessary care and stress [1, 13]. Understanding and addressing difficulties families face as they bring their child home with new needs and challenges is an important part of high quality compassionate care. The need for an efficient and effective process is well accepted; however, how this is best accomplished remains unclear. In 2009 multiple stakeholders from American College of Physicians, Society of General Internal Medicine, Society of Hospital Medicine, American Geriatrics Society, American College Of Emergency Physicians, and Society for Academic Emergency Medicine developed a transitions of Care Consensus statement in which they identified standards for care transitions, but did not offer clear recommendations on how to accomplish the goals of timely, standardized communication that included patient and family involvement [14].

Post discharge phone calls have been used as a strategy to improve communication with patients including more prompt recognition of problems and clarifying recommendations [5, 7, 8, 10–12, 15, 16]. As a result, post discharge phone calls have been found to improve patient satisfaction [12]. On the other hand, some providers feel that assigning a nurse to place the phone calls represents a diversion of a needed resource and is not of value [16]. In fact, this was debated in our unit. It is difficult to assign a dollar value to patient satisfaction or resolution of problems; individual units must assess their resource availability in determining whether to undertake the process. The reality is that, on average, only one patient every 3 days was called. We submit that 20 min ever three days is a cost-effective and worthwhile investment when we consider the number of problems solved.

It was unclear whether these type of phone calls were effective in reducing readmission to the hospital [11, 15, 17]. Cost avoidance post hospital discharge including readmission is a significant concern and has been addressed by a variety of care initiatives focused on improved communication with patients [18-20]. Of note, on our rehabilitation unit, hospital readmission is not generally a significant concern as patients have often had a prolonged period of medical stability during their rehabilitation course prior to discharge.

Evaluating patient and family needs during transitions of care can be particularly challenging from a rehabilitation perspective. Data that are more easily available such as re-admission rates, procedures performed or infection rates do not necessarily reflect function in the home setting or caregiver satisfaction with the level of support provided as they learn to care for a newly disabled family member. Families of children with brain injuries have reported feelings of abandonment and increased stress during the transition from inpatient to outpatient rehabilitation [1]. Often in our practice, unmet needs would not be identified until the follow up rehabilitation medicine visit 4–6 weeks post discharge, if it occurred, resulting in a delay in assisting a family in obtaining needed care. Parent perception of unmet need has been associated with increased caregiver burden [3, 4]. Unmet needs are common in children with brain injury a common diagnosis in inpatient pediatric rehabilitation, especially in the first months following injury [1, 3, 4]. This is similar to adult medical and surgical patient populations and represents a wide spread concern [20]. This pilot study did identify a significant complexity of post-discharge care in our patient population as well as an area of need as families were having difficulty understanding recommendations and obtaining needed care.

Our study did have several limitations. As a pilot study our sample size was small. We did not analyze our sample based upon demographic characteristics such as age, race, ethnicity, primary language or socioeconomic status, all of which could affect access to resources. We did not use a standardized instrument for the phone call. We were not able to survey all of the families cared for on our unit as we were limited to those who responded to the phone call. Although the overall patient satisfaction scores for the unit improved after the implementation of the phone calls, there is no direct link.

## Conclusions

Based upon the results of this pilot study we conclude that our patients and families do have complex needs at the time of their discharge from inpatient rehabilitation. There is often confusion regarding recommendations and difficulty obtaining needed care. We also found that a post discharge phone call from an experienced rehabilitation nurse was able to address this confusion and assist families in this challenging transition. This cost effective intervention would be applicable to many patient populations facing difficult transitions with complex discharge needs. Further investigation into this intervention would be warranted.

This material was presented in part at the International Forum on Safety and Quality in London, England 2013.

## Declarations

The publication costs for this article were covered in full by a grant from the Colorado Physician Insurance Company (www.copic.com) to Philip F. Stahel, MD.

COPIC had no influence on authorship or scientific content of this article.
